# Mitigating SO_2_ Poisoning Effect in Electrochemical CO_2_ Reduction with Transition-Metal Single-Atom Molecular Catalysts

**DOI:** 10.1021/acs.energyfuels.6c02595

**Published:** 2026-08-08

**Authors:** Yiqing Wu, Chang Liu, Prajeet Oza, Sungho Jeon, Erika Ortega Ortiz, Yuanyuan Li, Juliane Weber, Eric A. Stach, Guoxiang Hu, Zili Wu

**Affiliations:** † Chemical Sciences Division, 6146 Oak Ridge National Laboratory, Oak Ridge, Tennessee 37830, United States; ‡ School of Materials Science and Engineering, 1372 Georgia Institute of Technology, Atlanta, Georgia 30332, United States; § Department of Materials Science and Engineering, 209856 University of Pennsylvania, Philadelphia, Pennsylvania 19104, United States; ∥ School of Chemistry and Biochemistry, Georgia Institute of Technology, Atlanta, Georgia 30332, United States

## Abstract

The electrochemical CO_2_ reduction reaction (CO2RR) offers a promising approach for converting captured CO_2_ into valuable chemicals and fuels. However, CO_2_ streams from industrial sources often contain SO_2_ impurities, which compromise the performance and stability of many electrocatalysts. Herein, we report the impact of SO_2_ on immobilized single-atom transition-metal (TM) centers—cobalt, nickel, and copper phthalocyanines (TM-PCs) on graphene. We show how the interaction between metal centers and SO_2_ impurities under CO2RR conditions affects the catalytic response. Among them, the Co-Pc/graphene catalyst demonstrates notable resistance to SO_2_ poisoning in membrane electrode assembly flow cells, while the other two TM-PCs deactivate. In situ X-ray absorption spectroscopy combined with density functional theory calculations indicates excellent structure stability of the Co-Pc/graphene catalyst and its favorable binding affinity for CO2RR intermediates over SO_2_-derived species. In contrast, both Cu-Pc/graphene and Ni-Pc/graphene catalysts exhibit severe degradation of the metal-N_4_ coordination structure and exhibit higher binding affinity toward SO_2_ species, leading to substantial activity loss in CO2RR. This work highlights the potential of tuning the metal centers of molecular catalysts to enhance impurity tolerance in electrochemical systems.

## Graphical abstract

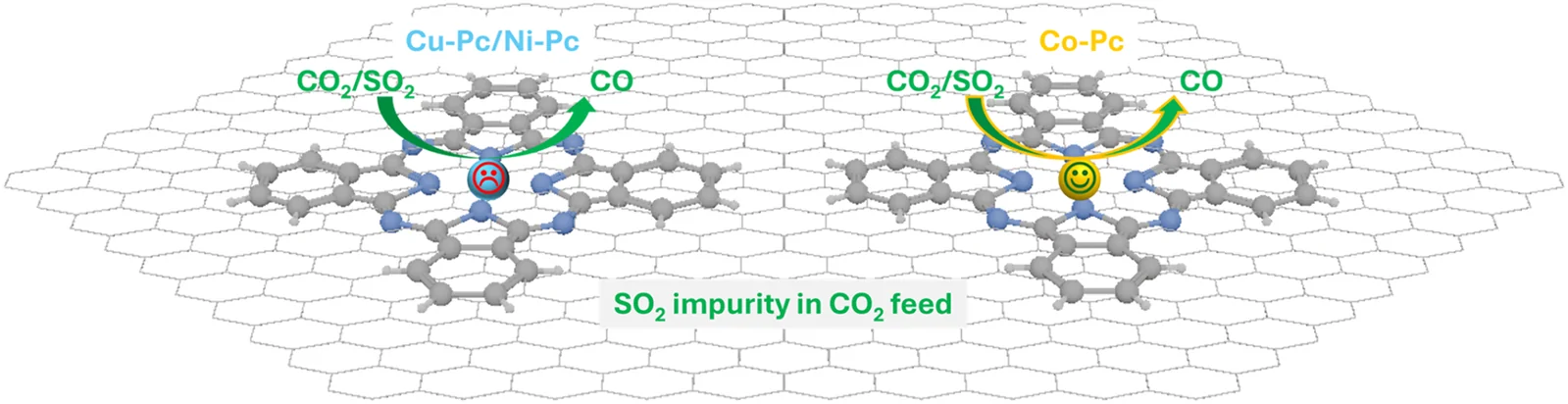

## Introduction

The electrochemical CO_2_ reduction reaction (CO2RR) has emerged as a promising route to convert waste CO_2_ to value-added hydrocarbon feedstocks.^1–3^ Extensive research effort has been devoted to developing highly selective and active electrocatalysts, as well as understanding the structure–activity correlation of active sites.^4–13^ However, most studies to date have been conducted under idealized conditions using pure CO_2_ feed, neglecting the influence of gas-phase impurities and simplifying mechanistic investigations. In contrast, real-world CO_2_ sources—such as industrial exhaust—typically contain various impurities, including sulfur dioxide (SO_2_), nitrogen oxides (NOx), and volatile organic compounds (VOCs), all of which can adversely impact catalyst performance and reduce electrolysis efficiency.^14–16^ Lee et al. have reported that SO_2_ concentration in the industrial exhaust can reach 3000 ppm, which can be further reduced to 40–135 ppm using various SO_2_ scrubbing processes.^17^ Given the intensive energy input in gas purification steps,^18^ a critical challenge for practical CO_2_ electrolysis is the development of robust electrocatalysts that can maintain stability and activity in the presence of contaminants, thereby enabling long-term CO_2_ conversion. One promising approach is to design electrocatalysts that are both selective for CO_2_ reduction and inert toward the reduction of impurity gases.

The interaction strength between impurity molecules and catalytically active sites serves as a key structural descriptor for impurity tolerance: stronger binding of impurity gases like SO_2_ can block active sites, change the catalyst structures, promote side reactions, and reduce the faradaic efficiency of CO2RR.^15,19,20^ Previous works have shown that the variation of binding energy between SO_2_ and metal active sites (such as Cu, Ag, and Sn) could lead to reversible or irreversible poisoning effects in CO2RR.^16,21^ However, most of these studies focused on metal nanoparticles,^16,21,22^ whose heterogeneous nature often obscures the understanding of how contaminants interact with and impact the metal active sites. In contrast, single-site catalysts offer an ideal platform for mechanistic study because of their relatively uniform active centers,^23,24^ allowing the isolation and clear examination of interactions between active sites and impurity species. Among the single-site catalysts, well-defined transition-metal phthalocyanine (TM-Pc) molecular catalysts have recently emerged as promising candidates for CO2RR, leading to different products such as CO and methanol (among others), with the product depending on the metal centers and the modification of the Pc structure.^6,17,18^ However, little is known about how they behave in the presence of impurities during the CO2RR reaction.

Herein, we report the influence of SO_2_ on the performance of CO2RR over TM-Pc molecular catalysts with varied metal centers—cobalt, copper, and nickel. The electronic structure of metal centers in TM-Pcs plays a pivotal role in modulating both activity and selectivity. However, under reaction conditions, TM-Pcs are prone to aggregation, leading to deteriorated CO2RR performance. In addition, the applied reduction potential can degrade the structural integrity of active sites, including both the metal sites and surrounding ligands, resulting in decreased CO2RR activity and selectivity.^6,25^ To improve molecular dispersion and catalyst stability, these TM-Pcs were immobilized on a conductive single-layer graphene support via strong π–π interactions.^25–27^ Our experiments reveal that among these three TM-Pcs, Co-Pc/graphene exhibits exceptional resistance to SO_2_ poisoning during electroreduction of CO_2_ to CO, highlighting its potential for impurity-resilient catalysis. In situ X-ray absorption spectroscopy (XAS) under CO2RR conditions combined with ex situ scanning transmission electron microscopy (STEM) demonstrated high structure stability of the Co-Pc active sites in the presence of SO_2_. Complementary density functional theory (DFT) calculations reveal that only the Co-Pc active sites preferentially bind to CO2RR intermediates rather than SO_2_-derived species, whereas the Cu-Pc and Ni-Pc show stronger binding affinity toward SO_2_-derived species than CO2RR intermediates, providing mechanistic insights into the relationship between electronic structure and impurity tolerance of TM-Pc catalysts. This study offers a strategy for the rational design of SO_2_-tolerant electrocatalysts and advances our understanding of how transition-metal electronic properties govern impurity resistance in electrochemical systems.

## Results and Discussion

### Morphology and Composition of Graphene-Supported TM-Pc Cathodes

Immobilization strategies to integrate molecular catalysts with heterogeneous materials have been shown to be important to improve the dispersion of molecular catalysts and enhance their catalytic stability. Two-dimensional (2D) graphene possesses high surface area and excellent electronic conductivity, offering an ideal support to disperse and stabilize TM-Pcs molecular catalysts.^27^ We incorporated TM-Pcs molecular catalysts onto 2D graphene supports via the π–π stacking interaction between Pc ligands and the graphene support (Scheme 1) to achieve atomic dispersion of TM-Pcs on the single-layer graphene. The homogeneously dispersed TM-Pcs in dimethylformamide (DMF) were mixed with the single-layer graphene powder by sonication, producing TM-PCs-loaded graphene composite catalyst (TM-Pc/G) with 1.3 wt % metal loading on the graphene. Scanning transmission electron microscopy (STEM) (Figure 1A) shows the 2D nature of the graphene support, and the presence of wrinkles and bright lines of contrast indicates that the graphene is very thin, which improves its electronic conductivity as a support material. Upon TM-Pc loading, the TM-Pc/G catalysts display a homogeneous distribution of metal single sites across the 2D graphene support (Figure 1B–1D) without aggregation, indicating atomic dispersion of all the TM-Pc catalysts promoted by the single-layer graphene.

**Figure 1:**
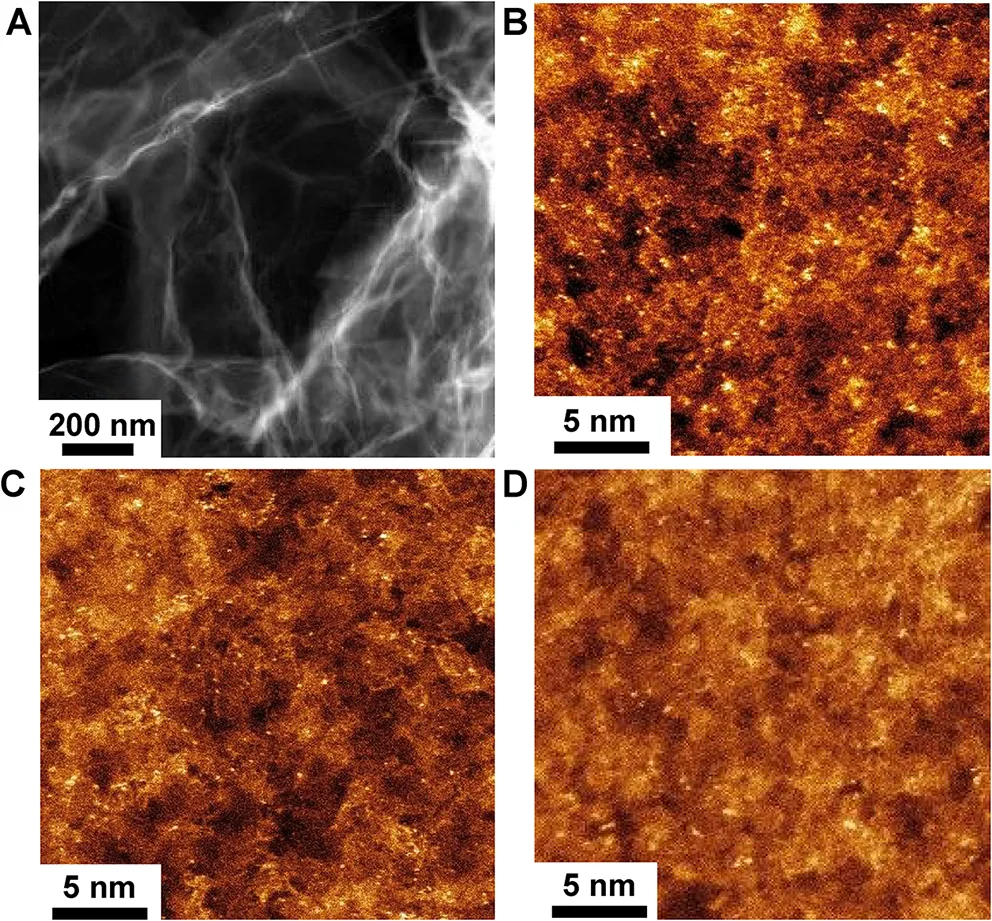
High-angle annular dark-field (HAADF) STEM images of single-layer graphene (A), Co-Pc/G (B), Ni-Pc/G (C), and Cu-Pc (D).

**Scheme 1:**
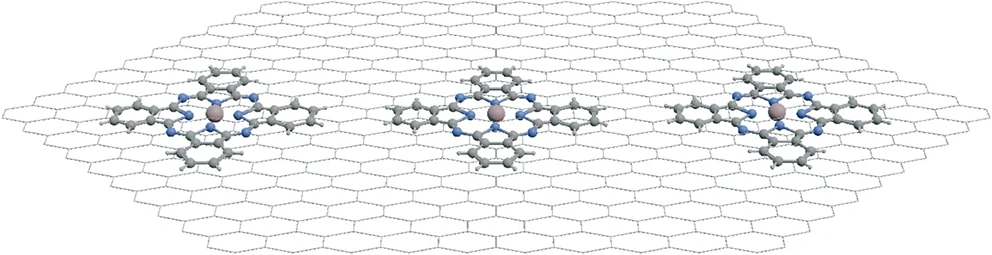
TM-Pc Deposited on Single-Layer Graphene via π–π Interaction for Uniform Coverage

### CO2RR Activity

Co-Pc/G, Ni-Pc/G, and Cu-Pc/G catalysts were evaluated for CO2RR using a membrane electrode assembly (MEA) reactor, as depicted in Scheme 2. A humidified gas mixture of CO_2_/argon at a 2:1 volume ratio was continuously fed into the cathode side at a flow rate of 30 mL/min, and a 0.1 M KHCO_3_ aqueous electrolyte was circulated on the anode side at a flow rate of 10 mL/min using a peristaltic pump. Chronoamperometry tests were performed at a constant potential of 3.0 V, and the reaction products were analyzed with in-line gas chromatography (GC). For all three catalysts, carbon monoxide (CO) and hydrogen (H_2_) are the only products. It is worth noting that the catalyst loading was kept the same for all three TM-Pc/G catalysts, with a TM-Pc/G loading of 0.1 mg cm^–2^ or a TM-Pc loading of 0.013 mg cm^–2^ on the electrode. Given the same loading, the partial current density represents the intrinsic activity of these TM-Pc/G catalysts.

**Scheme 2:**
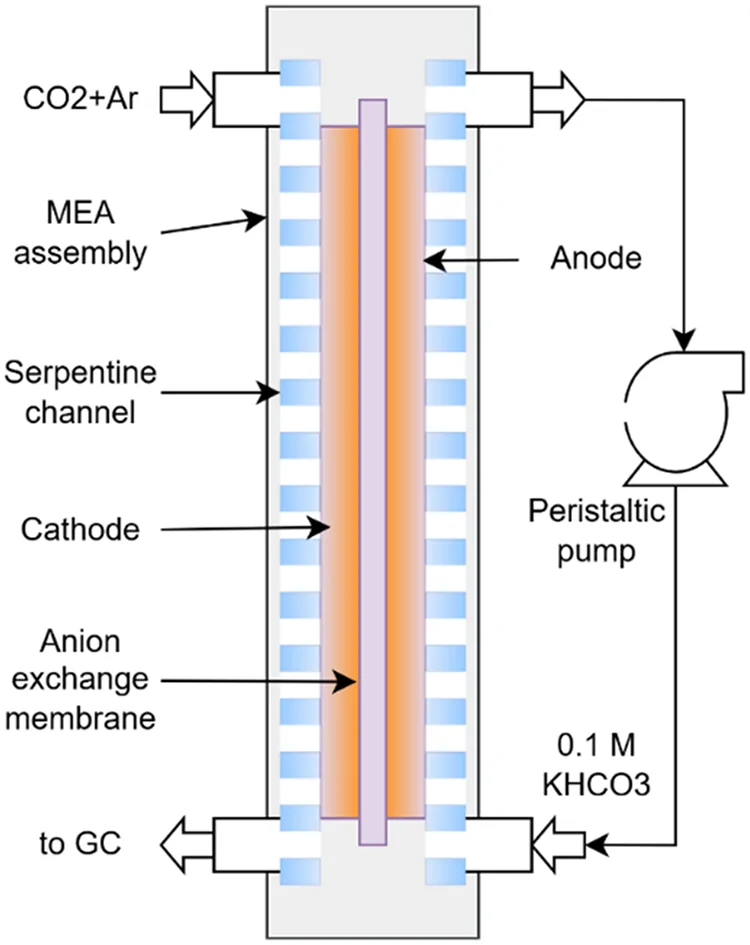
MEA Device for SO_2_ Impacts on CO_2_RR over TM-Pc/G Catalysts

The CO partial current density of Co-Pc/G in Figure 2A (orange bars) remains relatively stable at 10 mA cm^–2^ over the reaction time of 30 min, which was repeated one time to confirm its reproducibility, as shown in Figure S1. Subsequently, a 10 mL/min SO_2_ flow (200 ppm of SO_2_/Ar) was combined with the 20 mL/min CO_2_ flow and fed to the cathode at an SO_2_ concentration of ∼67 ppm, replacing the 10 mL/min inert Ar flow in the feed. The SO_2_ exposure leads to a slight increase in the CO partial current density of the Co-Pc/G catalyst at time on stream (TOS) of 10, 20, and 30 min, as shown in Figure 2A (green bars). In contrast, upon exposure to the same SO_2_ contaminant, both Ni-Pc/G and Cu-Pc/G catalysts show a dramatic decrease in CO partial current density from 2.1 to 0.2 mA cm^–2^ and 2.1 to 0.3 mA cm^–2^, respectively, at a TOS of 10 min, reflecting a loss of 91% and 85%, respectively, as shown in Figure 2C,E (orange bars and green bars).

**Figure 2:**
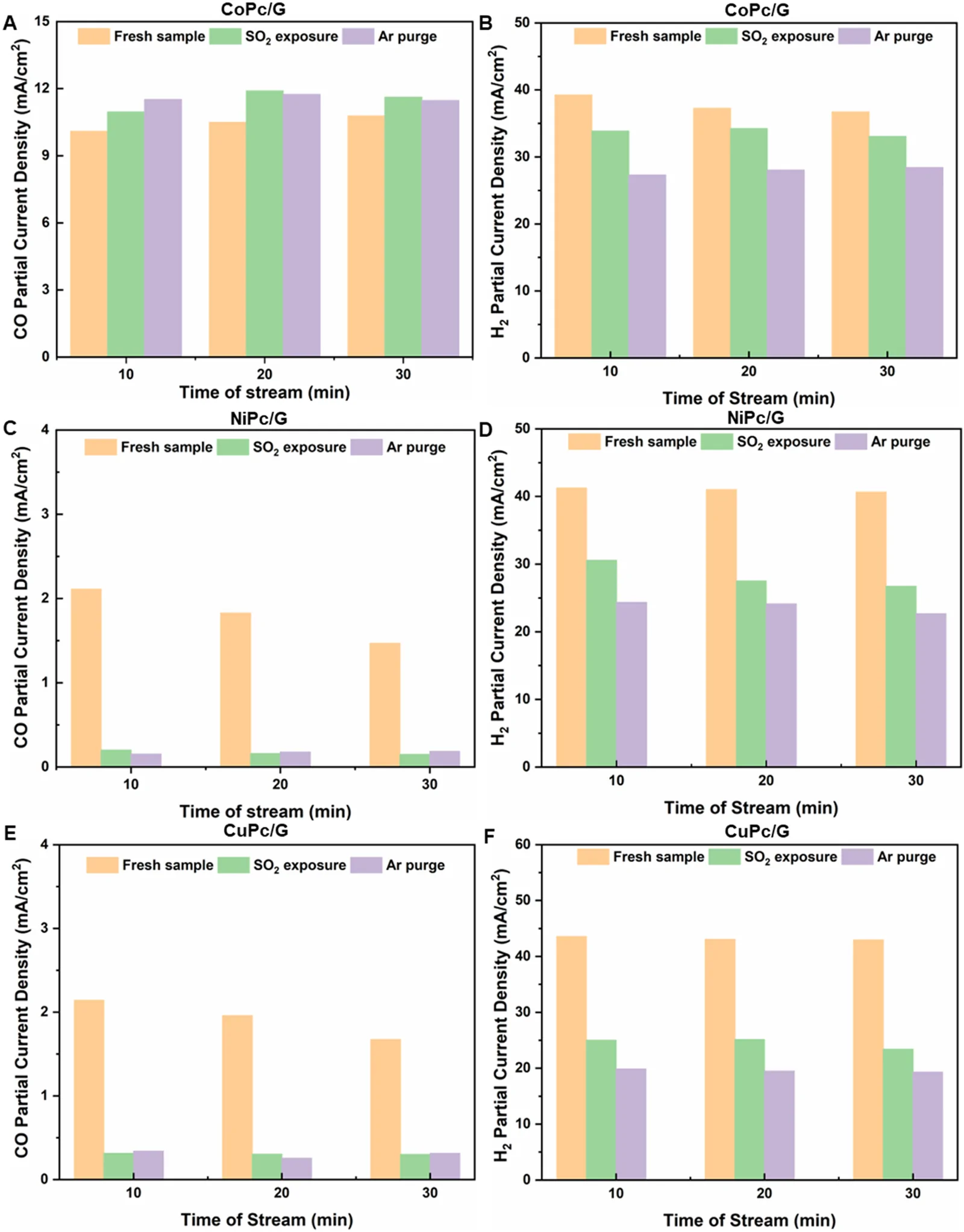
CO partial current density for Co-Pc-G (A), Ni-Pc-G (B), and Cu-Pc-G (C) catalysts and H_2_ partial current for Co-Pc-G (D), Ni-Pc-G (E), and Cu-Pc-G (F) catalysts during CO2RR reaction at three conditions: fresh samples, SO_2_ exposure, and Ar purge.

To assess whether these adverse impacts of SO_2_ on the Ni-Pc/G and Cu-Pc/G catalysts are reversible, the cathode gas feed was switched back to the CO_2_/Ar mixture (20 mL/min of CO_2_ + 10 mL/min of Ar). As shown in Figure 2C,E (purple bars), the SO_2_-poisoned Ni-Pc/G and Cu-Pc/G catalysts exhibit no recovery in CO partial current densities. As such, the persistent SO_2_ poisoning impacts for these two catalysts cannot be mitigated by simply stopping the flow of the SO_2_ contaminant. In contrast, the Co-Pc/G sample shows no changes after the removal of SO_2_ (Figure 2A).

Our results clearly indicate the superior stability of the Co-Pc/G catalyst against SO_2_ poisoning compared to the Ni-Pc/G and Cu-Pc/G catalysts. As the molecular metal-N_4_ ligand structure is essential for the high CO2RR activity of TM-Pcs,^6^ STEM imaging was conducted after the electrochemical testing to elucidate the impacts of SO_2_ exposure on the structural integrity of the TM-Pc catalytic sites, particularly the Ni-Pc/G and Cu-Pc/G catalysts. As presented in Figure 3, all TM-Pc/G catalysts retained well-dispersed single-site metal centers, with no evidence of TM-Pc aggregation. The retained atomic dispersion is consistent with the strong π–π interaction between the anchored TM-Pc and the graphene, preventing the aggregation of TM-Pcs. In addition, this clearly rules out leaching of metal centers from the metal-N_4_ coordination structure caused by total destruction of the metal-N_4_ coordination structure, which would lead to aggregation of metal centers. Therefore, the adverse impacts of SO_2_ on the Ni-Pc/G and Cu-Pc/G catalysts must have other origins. In previous studies, SO_2_ has been reported to adsorb on the metal centers, thereby blocking the access of CO_2_ intermediates to the active sites or disrupting the electronic properties of the metal-N_4_ ligands, leading to deteriorated CO2RR performance,^16,22^ which will be further elaborated in the following sections.

**Figure 3:**
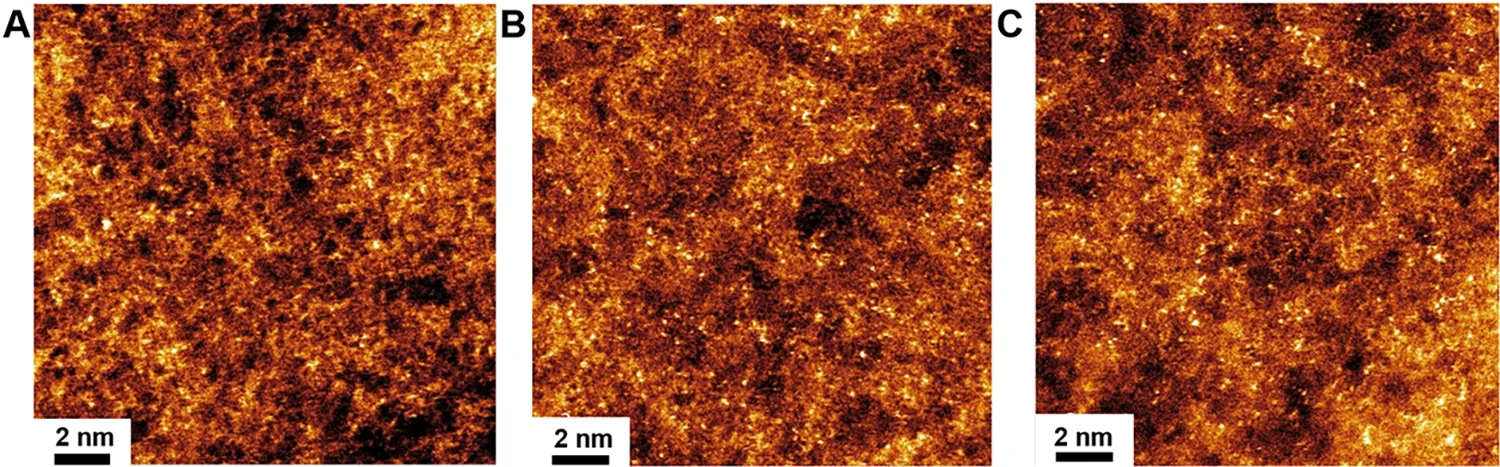
STEM images of spent TM-Pc/G catalysts after reaction for Co-Pc/G (A), Ni-Pc/G (B), and Cu-Pc/G (C).

For the competitive HER reaction, the partial current densities of H_2_ remain relatively stable at around 40 mA cm^–2^ across all TM-Pc/G catalysts, indicating a similar HER activity among these three catalysts, as shown in Figure 2B,2D,F (orange bars). Upon exposure to the SO_2_ contaminant, H_2_ partial current density shows a moderate loss for these catalysts, approximately 14% for Co-Pc/G, 25% for Ni-Pc/G, and 42% for Cu-Pc/G at a TOS of 10 min, which shows no recovery at longer TOS, as shown in Figure 2B,D,F (green bars). This activity loss in HER clearly indicates that SO_2_ can block active sites for both the CO2RR and HER, particularly the Ni-Pc/G and Cu-Pc/G catalysts. These trends corroborate the stronger resistance to SO_2_ of the Co-Pc catalyst on graphene. Reversing back to the SO_2_-free CO_2_/Ar feed even leads to a slight decrease in the H_2_ partial current density for all three catalysts, reflecting persistent negative impacts of SO_2_ on the HER activity.

These results over the CO2RR and HER suggest that SO_2_ exposure deteriorates the HER activity for Co-Pc/G but shows no impact on its CO2RR activity. However, for both Ni-Pc/G and Cu-Pc/G catalysts, the adverse impacts of SO_2_ are much more severe on the CO2RR than on the HER. As a result, the CO FE slightly increases from 20% to 24% upon SO_2_ exposure at a TOS of 10 min for Co-Pc/G, as shown in Figure S2. In comparison, the CO FE decreases from 4.9% to 0.7% for Ni-Pc/G and from 4.7% to 1.1% for Cu-Pc/G at a TOS of 10 min due to SO_2_ exposure.

### XAS Analysis

To reveal impacts of SO_2_ on the local structure of metal centers, we performed in situ XAS measurements at the Co K edge and Cu K edge to monitor the structural evolution of catalytic centers during CO2RR over the Co-Pc/G and Cu-Pc/G catalysts, both in the absence and presence of sulfur-related impurities. Because the MEA reactor setup is not available at the beamline facility, we employed an H-type cell to elucidate the impacts of SO_2_ on the structure and local coordination of metal centers in the Pc structure. H-type cells differ substantially from MEA reactors regarding local pH, microenvironment, mass transport, and maximum current density, which often lead to different catalyst activity and product selectivity. However, since both configurations inherently share the same reaction pathway, intermediates, and intrinsic activity, the impacts of SO_2_ can be translated from the H-type cell to the MEA reactor. To ensure a reliable correlation, the H-type cell electrodes were fabricated using the same method as those used in the MEA reactor. The working electrode—carbon paper loaded with TM-Pc/G catalysts—was biased at potentials −0.7, −0.9, and −1.1 V in 0.1 M KHCO_3_ aqueous electrolyte while being simultaneously illuminated with the X-ray beam to collect absorption data. As SO_2_ readily reacts with aqueous KHCO_3_ to form K_2_SO_3_, we added 0.025 M K_2_SO_3_ into the 0.1 M KHCO_3_ electrolyte to simulate the SO_2_ contaminant while mitigating the potentially hazardous condition caused by SO_2_ gas flow at the synchrotron beamline. Regarding the interaction with electrodes, the sulfite (SO_3_^2–^) ions are anticipated to be different from SO_2_ gas molecules in terms of local pH and mass transfer efficiency as they are dispersed in the liquid KHCO_3_ electrolyte. To verify whether K_2_SO_3_ can be employed to simulate the SO_2_ contaminant in aqueous solutions, an H-type cell was separately tested for Co-Pc/G and Cu-Pc/G catalysts before going to the beamline, in which 0.1 M KHCO_3_ was used as the electrolyte. CO2RR and HER activity were collected first with the fresh catalysts. After that, 0.025 M K_2_SO_3_ was added to the electrolyte, and the experiments were repeated after 30 min to ensure adequate exposure of electrodes to the added K_2_SO_3_ impurity. As shown in Figure S3A, the CO partial current density of the Co-Pc/G catalyst remains nearly unaltered or slightly improved upon the introduction of 0.025 M K_2_SO_3_ at −0.6, −0.7, and −0.8 V. In contrast, the Cu-Pc/G catalyst shows a clear decline in CO partial current density at −0.9, −1.0, and −1.1 V. It is worth noting that the Cu-Pc/G catalyst was tested under more negative potential than the Co-Pc/G catalyst due to its low CO2RR activity in the range of −0.6 to −0.8 V. For their HER activity, both catalysts show a substantial decrease. These trends are consistent with those observed using the MEA reactor, confirming that K_2_SO_3_ can be used to simulate the SO_2_ contaminant in the XAS study.

For the Co-Pc/G catalyst, the XANES and EXAFS spectra (Figure 4A,B) showed minimal change under different potentials and after the K_2_SO_3_ impurity introduction. The XANES spectral features of Co-Pc/G are quite different from those of CoO, and the EXAFS spectra of Co-Pc/G only show a pronounced Co–N path with a bond length of ∼1.88 Å (Table S1 and Figure S4). The absence of Co–Co scattering implies no sintering of metal centers from Co-Pc/G during CO2RR reaction either before or after K_2_SO_3_ introduction, consistent with the STEM imaging. These findings corroborate well with the strong SO_2_ tolerance observed in electrochemical tests and ex situ STEM imaging of Co-Pc/G.

**Figure 4:**
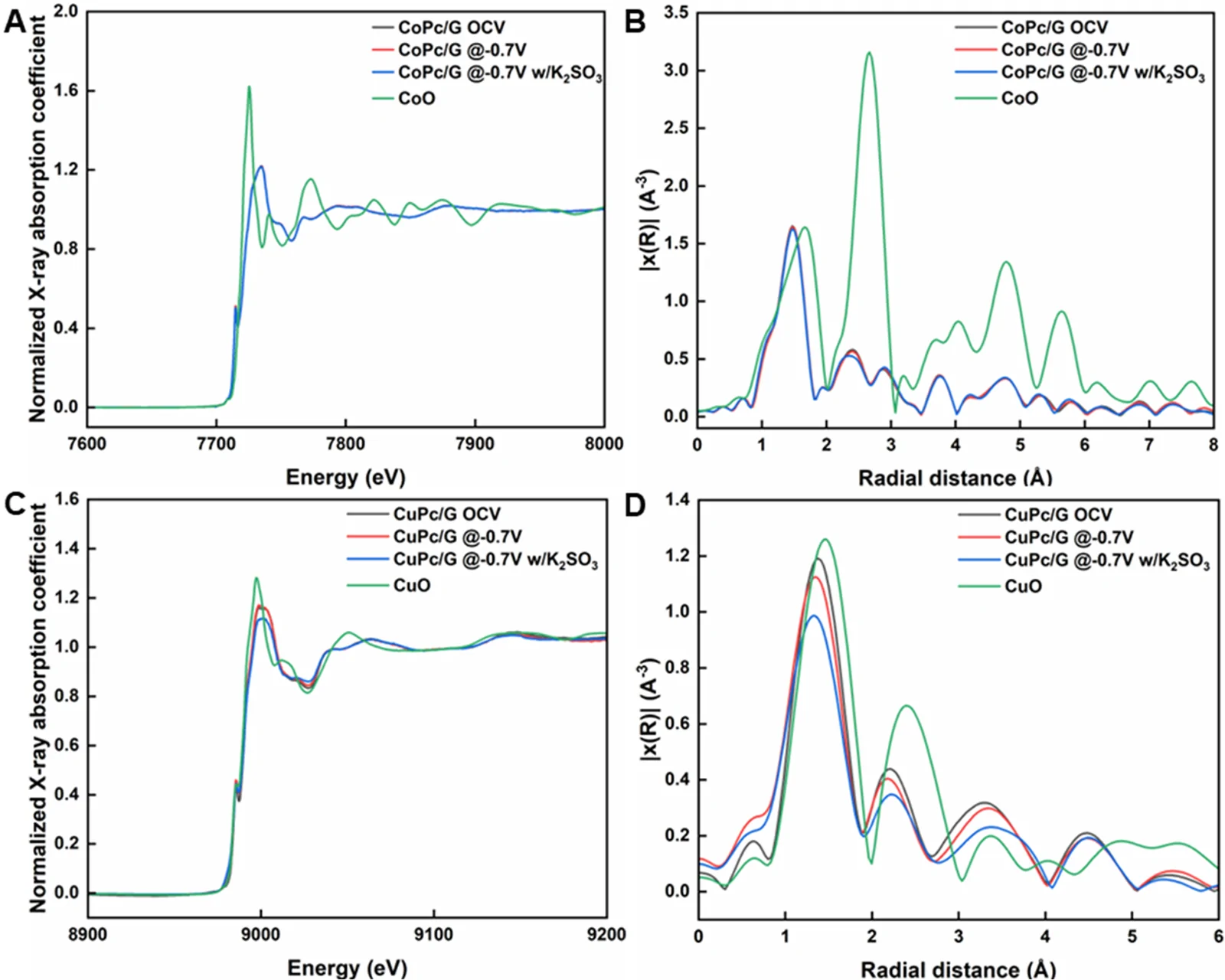
In situ Co K edge (A) XANES and (B) R-space EXAFS spectra for Co-Pc/G, and (C) Cu K edge and (D) R-space EXAFS spectra for Cu-Pc/G.

Due to the limited beamtime, only the Cu-Pc/G catalyst was selected for the in situ XAS analysis to investigate whether its poor resistance against SO_2_ poisoning originates from distinct structural responses to impurities. In Figure 4C, Cu-Pc/G displayed clear spectral changes upon K_2_SO_3_ introduction under the same CO2RR condition. The XANES spectra (Figure 4C) exhibited decreased intensity of the maxima at about 9000 eV after K_2_SO_3_ introduction, suggesting altered electronic structure, likely due to sulfur species interacting with the metal sites. Same as the Co-Pc/G catalyst, the absence of Cu–Cu scattering in its EXAFS fitting (Figure 4D) implies no sintering of the Cu metal centers. However, the decrease of the peak at about 1.5 Å indicates an altered local atomic structure around Cu center (a loss of coordinating N ligands to the Cu center and/or distorted Cu–N geometry as shown in Table S2) in Cu-Pc/G upon exposure to the sulfur contaminant. Unlike the intact Co-Pc structure, this pronounced sulfur-induced local structural change in Cu-Pc/G is likely to disrupt the local electronic properties of the Cu metal center and consequently, deteriorate its CO2RR performance. In addition, such a local structural change is irreversible, as indicated by the lack of performance recovery for the Cu-Pc/G catalyst after reverting to SO_2_-free CO_2_/Ar feed (Figure 2), further underscoring the distinct interactions between sulfur species and Co-Pc/G versus Cu-Pc/G.

### DFT Simulations

To gain further mechanistic insight into the distinct SO_2_ tolerance of the three TM-Pc/G catalysts, we performed density functional theory (DFT) calculations to compare the relative binding energies of a key CO_2_RR intermediate (COOH), an HER intermediate (H), and representative sulfur-related species (SO_2_ and HSO_3_). COOH was selected because its formation is widely regarded as a key proton-coupled electron-transfer step in the reduction of CO_2_ to CO and is therefore a more catalytically relevant descriptor than molecular CO_2_ adsorption.^28^ HSO_3_ was included as a representative sulfur-derived species relevant to the SO_2_ dissolution in an aqueous electrolyte.

As shown in Figure 5, Co-Pc exhibits more negative binding energies for COOH and H than for the sulfur-derived species considered. Within the present computational model, this result suggests that competitive adsorption by sulfur-derived species may be less favorable at the Co–N_4_ center. In contrast, Ni-Pc and Cu-Pc exhibit weaker energetic discrimination between the CO_2_RR/HER intermediates and sulfur-related adsorbates, suggesting greater susceptibility to competitive adsorption by sulfur-derived species. These calculated trends provide a possible thermodynamic rationale for the maintained CO production on Co-Pc/G and the pronounced deactivation and poor recovery observed experimentally for Ni-Pc/G and Cu-Pc/G. The stronger calculated binding of H relative to the sulfur-derived species on Co-Pc may also be consistent with the comparatively smaller decrease in HER activity observed for Co-Pc/G. However, the static binding energies do not directly determine steady-state adsorbate coverage or reaction rates, which also depend on the applied potential, solvation, and kinetic barriers.

**Figure 5:**
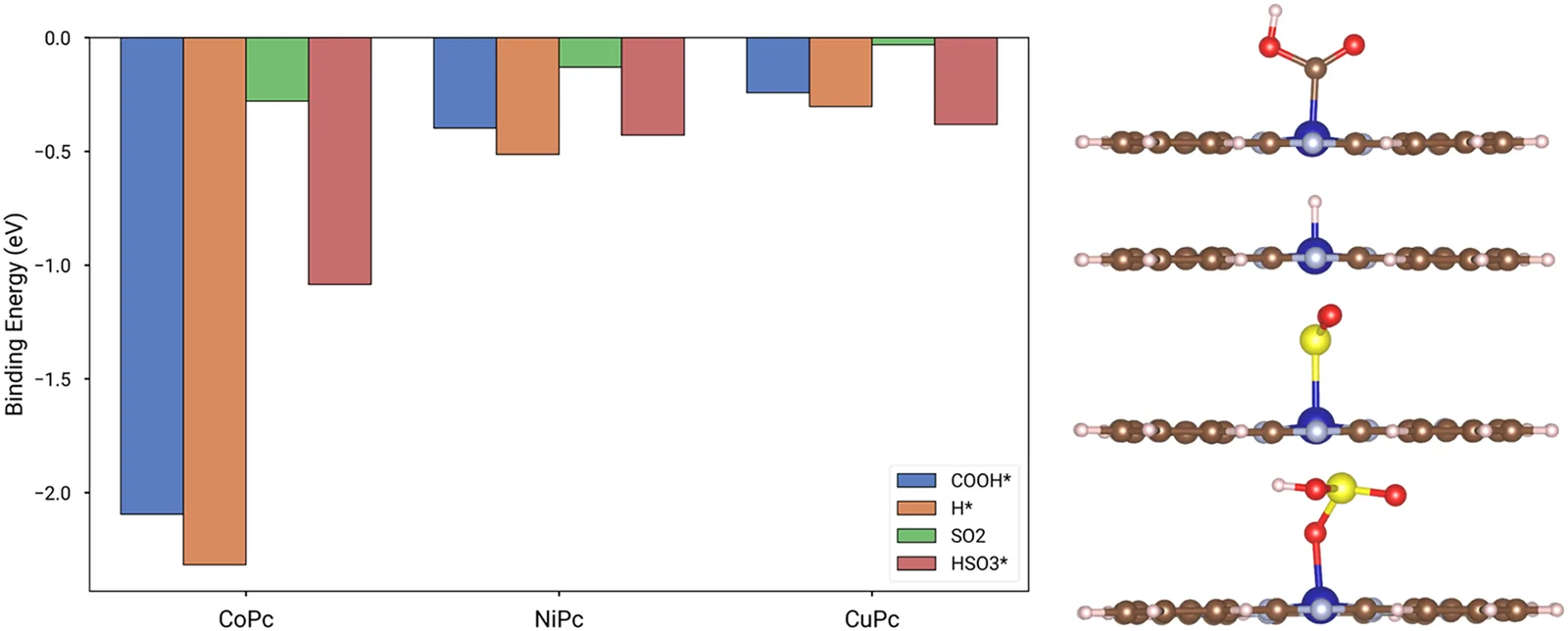
Zero-bias DFT-calculated binding energies of representative CO_2_RR/HER intermediates and sulfur-related adsorbates on Co-, Ni-, and Cu-Pc, together with the corresponding optimized structures. More negative binding energies indicate stronger binding.

Collectively, these results suggest that the identity and electronic structure of the central metal atom influence impurity tolerance by modulating the relative binding tendencies of the reaction intermediates and sulfur-derived species. Nevertheless, the electrochemical and in situ XAS measurements do not directly identify or quantify adsorbed sulfur species, and the calculated binding energies should not be regarded as direct evidence of the adsorbate identity or coverage under operating conditions. Rather, they serve as comparative thermodynamic descriptors that provide a possible rationale for experimentally observed trends.

## Conclusion

In this study, we examined how different metal centers influence SO_2_ poisoning in the CO2RR using graphene-supported TM-Pc model single-site catalysts by combining experiments, in situ XAS, and DFT simulations. Among the three TM-Pc catalysts, Co-Pc/G demonstrated exceptional SO_2_ tolerance with no loss in CO2RR activity, while Cu- and Ni-Pc/G displayed irreversible deactivation upon SO_2_ exposure. STEM imaging suggested that the metal centers stayed singly dispersed during the electrochemistry test. In situ XAS analysis showed that the Co-Pc structure remained intact upon exposure to SO_2_, while Cu-Pc exhibited significant local structural changes around the Cu center. DFT calculations confirmed that Co-Pc preferably binds to the CO2RR reaction intermediates over sulfur species, whereas both Ni-Pc and Cu-Pc exhibit comparable or even higher binding affinity to sulfur-derived species than the COOH intermediate. Overall, the distinct SO_2_ tolerance for the TM-Pc/G catalysts can be related to the stability of the coordinating ligands of the metal centers and the electronic properties of the metal centers that govern the binding affinity toward SO_2_-derived species vs CO_2_-derived reaction intermediates. This work provides a detailed mechanistic understanding of impurity-induced deactivation of single-atom molecular catalysts in the CO2RR and illustrates how tailoring the electronic structure of the catalytic centers can modulate the tolerance to impurities and thus the stability of the catalytic performance.

## Experimental Details

### Electrode Preparation

Cobalt­(II) phthalocyanine (Co-Pc, β form 97%), nickel­(II) phthalocyanine (Ni-Pc, 85%), and copper­(II) phthalocyanine (Cu-Pc, β form 90%) were purchased from Sigma-Aldrich. Single-layer graphene was purchased from ACS Material. Nafion solution (5 wt % in ethanol, XA-9) was purchased from Dioxide Materials. Ethanol (99.8% anhydrous) and *N*,*N*-dimethylformamide (DMF, 99.8% anhydrous) were purchased from Sigma-Aldrich. Carbon paper (Sigracet 39 BB) was purchased from Fuel Store with 5 wt % PFTE on one side for waterproofing, preventing water diffusion during CO2RR, and carbon black on the other side to enhance the electronic conductivity.

Single-layer graphene-supported TM-Pc catalysts were prepared by dissolving 1.5 mg TM-Pc in 30 mL DMF under sonication for 1 h. Subsequently, 10 mg single-layer graphene was added to the solution, followed by another hour of sonication to disperse TM-Pc on the single-layer graphene. The resulting suspension was centrifuged 3 times, washed with ethanol to remove residual DMF, and dried at 80 °C overnight in air. To prepare the ink solution, 2 mg of dried Co-Pc/graphene composite was mixed with 2 mL of ethanol and 100 uL of 5 wt % Nafion solution, followed by sonication for 1 h. The cathode was fabricated by drop-casting the ink solution onto the carbon black layer of the 5% PTFE waterproof carbon paper to achieve a catalyst loading of 0.1 mg of Co-Pc/graphene per cm^2^ of carbon paper, denoted as Co-Pc/G. Ni-Pc/G and Cu-Pc/G cathodes were prepared using the same procedure.

### Electrochemical Measurements

The CO2RR reaction was performed using a 5 cm^2^ membrane electrode assembly (MEA) purchased from Dioxide Materials. A commercial IrO_2_-deposited carbon paper with 4.8 mg of IrO_2_ loading per cm^2^ (Dioxide Materials) was used as the anode. An activated anion-exchange membrane (X37–50 GRADE RT, Dioxide Materials) was sandwiched between the TM-Pc/G cathodes and the commercial IrO_2_ anode with both catalyst layers facing each other. This sandwiched electrode-membrane structure was placed in the 5 cm^2^ MEA with the waterproof layers of the cathode and anode facing the serpentine channels of the MEA. To ensure that the cathode is fully covered by the anode, a cathode with a size of 2.2 × 2.2 cm was used, which is smaller than that of the anode at 2.4 × 2.4 cm.

The CO_2_/Ar (2:1 ratio) mixture flow at 30 mL/min was bubbled through DI water to humidify the feed stream before being introduced into the MEA cathode side. On the anode side, 0.1 M aqueous KHCO_3_ electrolyte (99.7% purity, Sigma-Aldrich) was continuously circulated using a peristaltic pump at 10 mL/min. The potential and current were controlled and recorded with an SP-300 potentiostat (BioLogic). The reactants and products were monitored using an SRI gas chromatograph equipped with both a flame ionization detector (FID) and a thermal conductivity detector (TCD).

### Scanning Transmission Electron Microscopy (STEM)

STEM images were collected by a NEOARM (200 kV) including a probe aberration corrector, with a 6c probe size and a 40 μm condenser aperture, yielding approximately 62.5 pA probe current.

### In Situ XAS Measurement

In situ Cu K edge XAS data were collected in the fluorescence mode at the QAS beamline (7-BM) of the National Synchrotron Light Source II (NSLS II), Brookhaven National Laboratory. The experiments were performed using a half-cell of a commercial in situ electrochemical cell (GDE XRD EC 1.75 mL, MSE Supplies). The TM-Pc/G cathode was pressed tightly to the half-cell’s window with the catalyst side facing the electrolyte and further sealed by a Kapton tape, which allows for X-ray transmission and fluorescence detection. As such, this half-cell functions as a single-compartment electrochemical cell, as depicted in Scheme S1 in Supporting Information. To enhance the fluorescence signal, the concentration of TM-Pc in the cathode was increased to 1 mg/cm^2^. Pt coil was employed as the anode and Ag/AgCl as the reference electrode. Aqueous 0.1 M KHCO_3_ electrolyte was circulated by a peristaltic pump at 10 mL/h, with 10 mL/min CO_2_ flow continuously bubbled through the electrolyte to saturate the electrolyte with CO_2_. A SP-300 potentiostat was used to apply the potential and collect current data. In a typical experiment, XAS data were first collected at −0.7, −0.9, and −1.1 V with the CO_2_-saturated electrolyte. After that, K_2_SO_3_ was added to the electrolyte to reach 0.025 M K_2_SO_3_, and the XAS experiment was repeated at the same potential to reveal the impacts of SO_2_. The collected XAS data were processed and analyzed via the IFEFFIT package.^29^

### DFT Calculation

Spin-polarized DFT calculations were performed using the Vienna Ab initio Simulation Package (VASP).^30,31^ The electron exchange-correlation was represented by the functional of Perdew, Burke, and Ernzerhof (PBE) of generalized gradient approximation (GGA).^32^ The ion-electron interaction was described with the projector augmented-wave (PAW) method.^33^ The plane-wave cutoff was set to 400 eV, and a conjugate gradient method was used to relax the geometry until the interatomic forces were less than 0.02 eV/Å. The vacuum separations in the *x* and *y* directions of the cobalt, nickel, and copper phthalocyanines are greater than 10 Å, and 20 Å in the *z* direction, to prevent unphysical interactions between periodic images. The Brillouin zone was sampled using a 3 × 3 × 1 Monkhorst–Pack *k*-point mesh.

Binding energies were calculated as *E*_bind_ = *E*_TM‑Pc‑X_ – *E*_TM‑Pc_ – *E*_X_, where *E*_TM‑Pc‑X_, *E*_TM‑Pc_, and *E*_X_ are the total energies of the adsorbate-bound TM-Pc complex, the isolated TM-Pc, and the corresponding isolated adsorbate reference, respectively. Here, X denotes COOH, H, SO_2_, or HSO_3_. A more negative binding energy indicates stronger and more energetically favorable binding. The present calculations were performed at zero applied bias without explicit solvation, electrolyte ions, electrode charging, interfacial electric fields, or potential- and pH-dependent proton/electron free-energy corrections. Therefore, the calculated binding energies should be interpreted as comparative thermodynamic descriptors of intrinsic binding tendencies rather than as electrochemical adsorption free energies under operating conditions.

## Supplementary Material



## Notes

Experimental section for detailed H-cell experiment and in situ XAS experiment, electrochemical experiment with reproducibility and stability (Figures S1–S3), and structural characterization of electrodes with in situ XAS (Figures S4 and S5) (Tables S1 and S2) (PDF)
