# Supplementary material for: Mitigating SO2 Poisoning Effect in Electrochemical CO2 Reduction with Transition-Metal Single-Atom Molecular Catalysts
Source: Energy Fuels. 2026 Aug 8;40(33):18478–86. doi: 10.1021/acs.energyfuels.6c02595 (PMC13501875; doi:10.1021/acs.energyfuels.6c02595)
Supplement: Supplementary file 1 [file ef6c02595_si_001.pdf]

Supplementary Information

Mitigating SO_2 poisoning effect in electrochemical CO_2 reduction with transition metal single-atom molecular catalysts

Yiqing Wu[a], Chang Liu[a*], Prajeet Oza[b], Sungho Jeon[c], Erika Ortega Ortiz[c], Yuanyuan Li[a], Juliane Weber[a], Eric A. Stach[c], Guoxiang Hu[b], Zili Wu[a*]

[a] Chemical Sciences Division, Oak Ridge National Laboratory, Oak Ridge, TN 37830, USA

[b] School of Materials Science and Engineering at Georgia Institute of Technology, Atlanta, GA 30332

[c] Department of Materials Science and Engineering, University of Pennsylvania, Philadelphia, Pennsylvania 19104, USA

Corresponding Author: Chang Liu, liuc5@ornl.gov; Zili Wu, wuz1@ornl.gov

CO2RR Faradaic efficiency

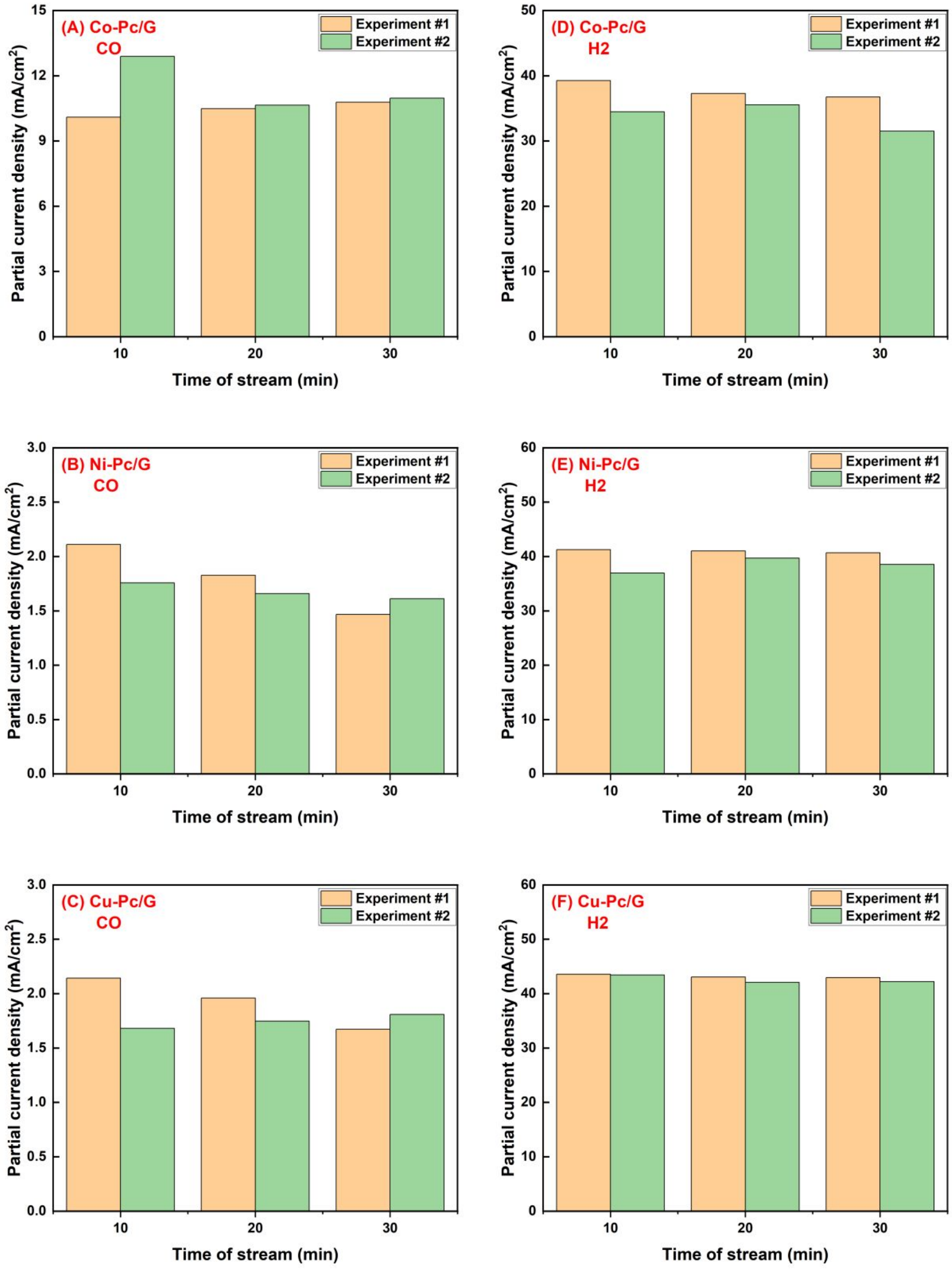

Figure S1. CO partial current density for Co-Pc-G (A), Ni-Pc-G (B) and Cu-Pc-G (C) catalysts and H_2 partial current for Co-Pc-G (D), Ni-Pc-G (E) and Cu-Pc-G (F) catalysts during two repeated CO2RR experiments.

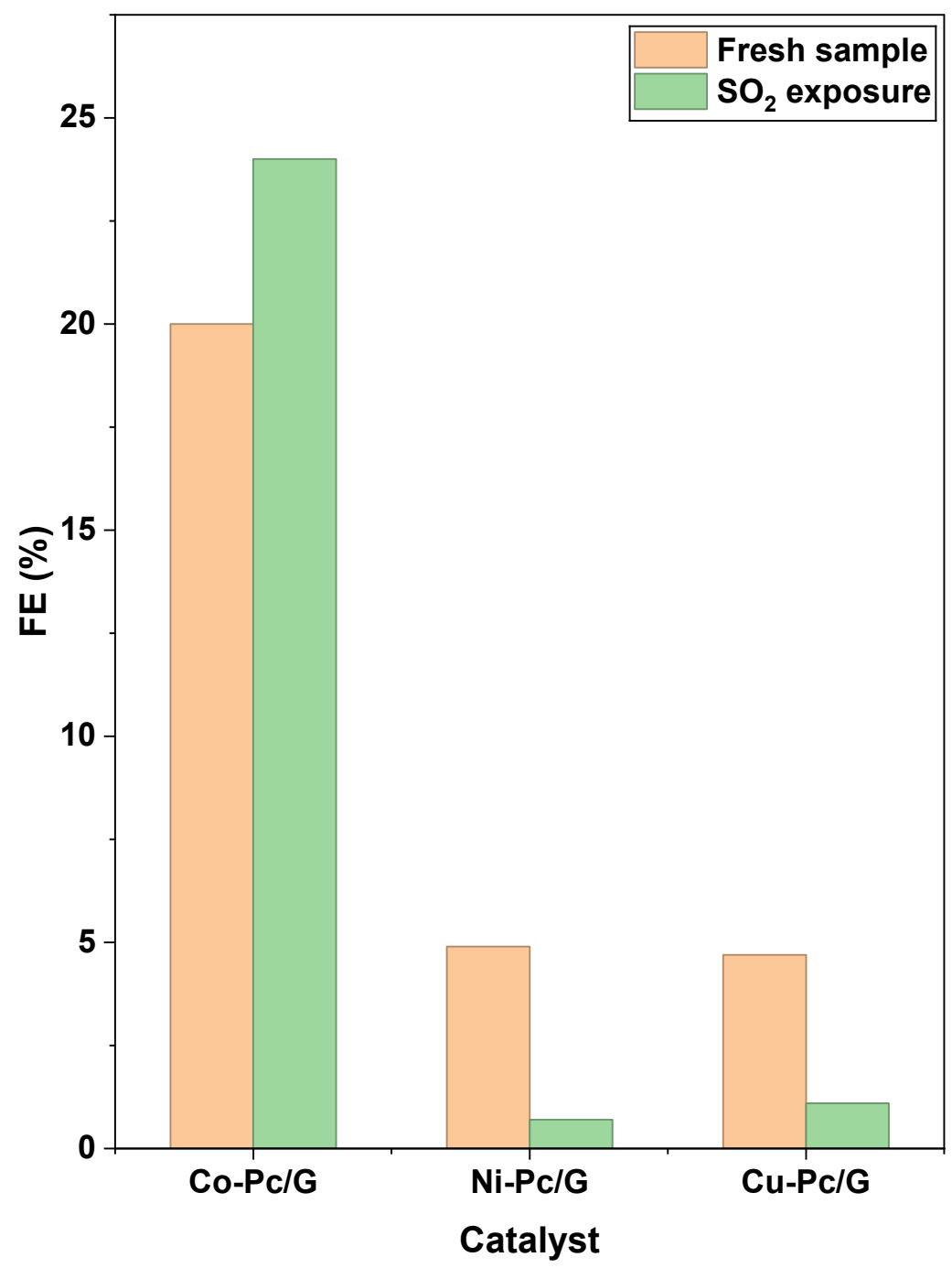

Figure S2. Faradaic efficiency (FE) for Co-Pc-G, Ni-Pc-G and Cu-Pc-G catalysts at TOS of 10 mins during CO2RR before and after SO2 exposure.

CO2RR performance over H-cell reactor

CO2RR reactions were carried out using a H-type cell reactor to elucidate the impacts of SO_2 on the catalytic performance of Co-Pc/G and Cu-Pc/G. As SO_2 readily reacts with water to form K_2SO_3, 0.005 M K_2SO_3 was added to the 0.1 M $KHCO_3$ electrolyte to study the impacts of SO_2. Co-Pc/G or Cu-Pc/G was loaded onto the 2x1 cm 10wt% PTFE waterproof carbon paper to fabricate cathodes with 1x1 cm of the carbon paper covered by the catalysts to reach 0.2 mg/cm^2 loading. High resolution STEM reveals that Co-Pc and Cu-Pc are atomically dispersed on the single layer graphene without aggregation. A commercial Ag/AgCl was employed as the reference electrode and placed in the same compartment with the cathode. A commercial IrO_2-deposited Ti mesh with 4.8 mg/cm^2 IrO_2 loading (Dioxide Material) was used as the anode and placed in the other compartment. An activated anion exchange membrane (X37-50 GRADE RT, Dioxide Material) was used to separate these two compartments. The potential and current were controlled and recorded by a SP-300 potentiostat (BioLogic). The reactants and products were monitored using an SRI gas chromatography equipped with both a flame ionization detector (FID) and a thermal conductivity detector (TCD).

Figure S1 shows the CO2RR performance of the Co-Pc/G cathode and the Cu-Pc/G cathode before and after the introduction of 0.005 M K_2SO_3. For Co-Pc/G catalyst, the current density of CO at -0.6 V is slightly lower upon introduction of K_2SO_3, but at both -0.7 and -0.8 V, the CO current density was slightly promoted by the addition of K_2SO_3. In contrast, the addition of 0.005 M K_2SO_3 led to a substantially lower H_2 current density at both -0.6 and -0.7 V. At -0.8 V, the H_2 current density shows no change before and after the introduction of K2SO3, which is likely due to the promoted HER activity at high potentials. For the Cu-Pc/G catalyst, the addition of 0.005 M K_2SO_3 consistently resulted in lower CO2RR activity at -0.9, -1.0 and -1.1 V. The

potentials used in the case of Cu-Pc/G catalysts are higher than those used for the Co-Pc/G catalyst, which is due to the much lower activity of Cu-Pc/G, making it essential to use a higher potential to examine the impacts of K_2SO_3 on its CO2RR activity. In addition, the H_2 current density of the Cu-Pc/G catalyst decreases substantially with the introduction of K_2SO_3 at these three potentials, clearly indicating a negative impact.

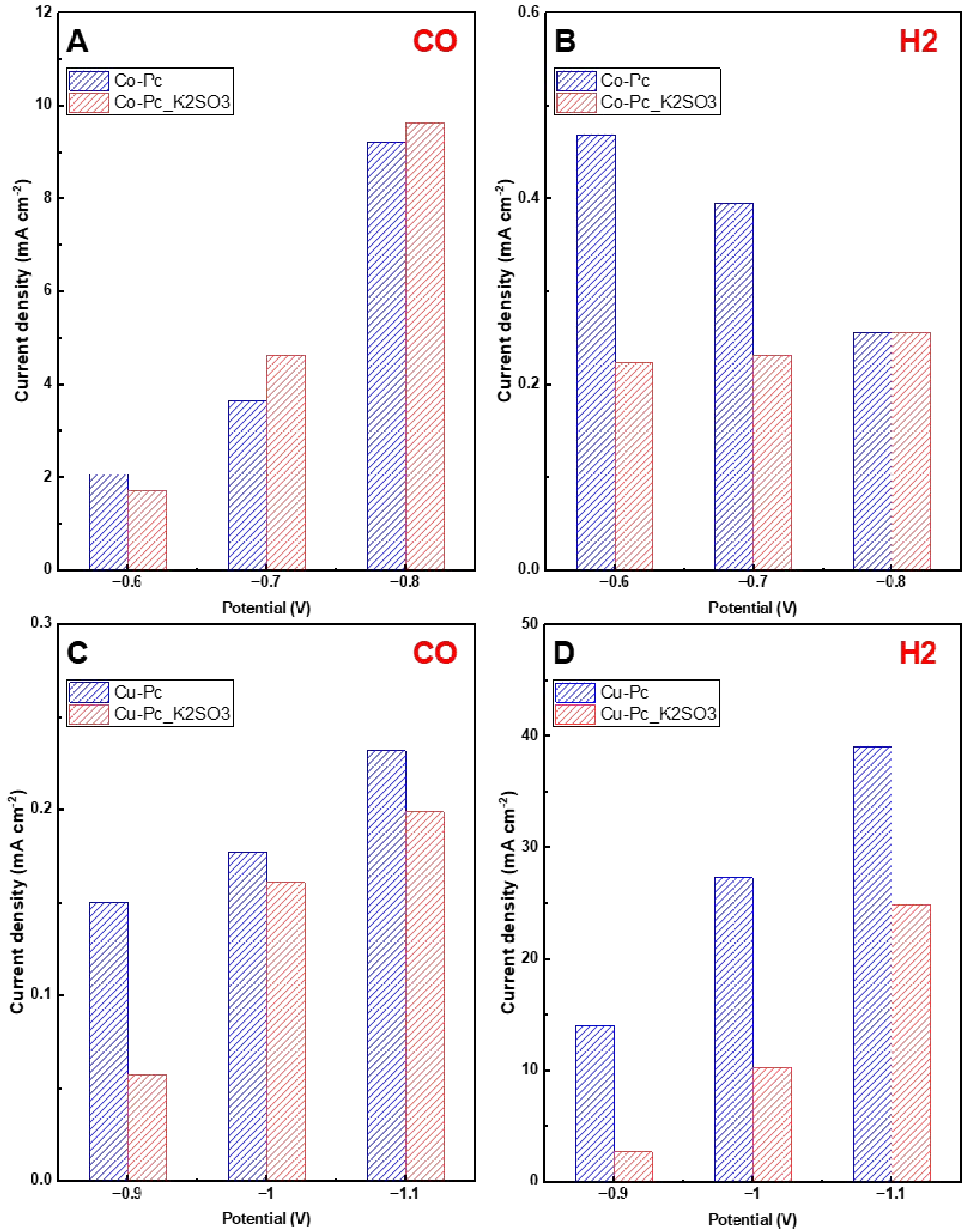

Figure S3. CO2RR performance over an H-cell before and after the introduction of 0.005 M K_2SO_3: (A) CO current density for Co-Pc/G, (B) H_2 current density for Co-Pc/G, (C) Co current density for Cu-Pc/G and (D) H_2 current density for Cu-Pc/G.

In situ X-ray adsorption spectroscopy

CO2RR reactions were carried out using a H-type cell reactor to elucidate the impacts of SO_2 on the catalytic performance of Co-Pc/G and Cu-Pc/G. As SO_2 readily reacts with water to form K_2SO_3, 0.005 M K_2SO_3 was added to the 0.1 M $KHCO_3$ electrolyte to study the impacts of SO_2. Co-Pc/G or Cu-Pc/G was loaded onto the 2x1 cm 10wt% PTFE waterproof carbon paper to fabricate cathodes with 1x1 cm of the carbon paper covered by the catalysts to reach 0.2 mg/cm^2 loading. High resolution STEM reveals that Co-Pc and Cu-Pc are atomically dispersed on the single layer graphene without aggregation. A commercial Ag/AgCl was employed as the reference electrode and placed in the same compartment with the cathode. A commercial IrO_2-deposited Ti mesh with 4.8 mg/cm^2 IrO_2 loading (Dioxide Material) was used as the anode and placed in the other compartment. An activated anion exchange membrane (X37-50 GRADE RT, Dioxide Material) was used to separate these two compartments. The potential and current were controlled and recorded by a SP-300 potentiostat (BioLogic). The reactants and products were monitored using an SRI gas chromatography equipped with both a flame ionization detector (FID) and a thermal conductivity detector (TCD).

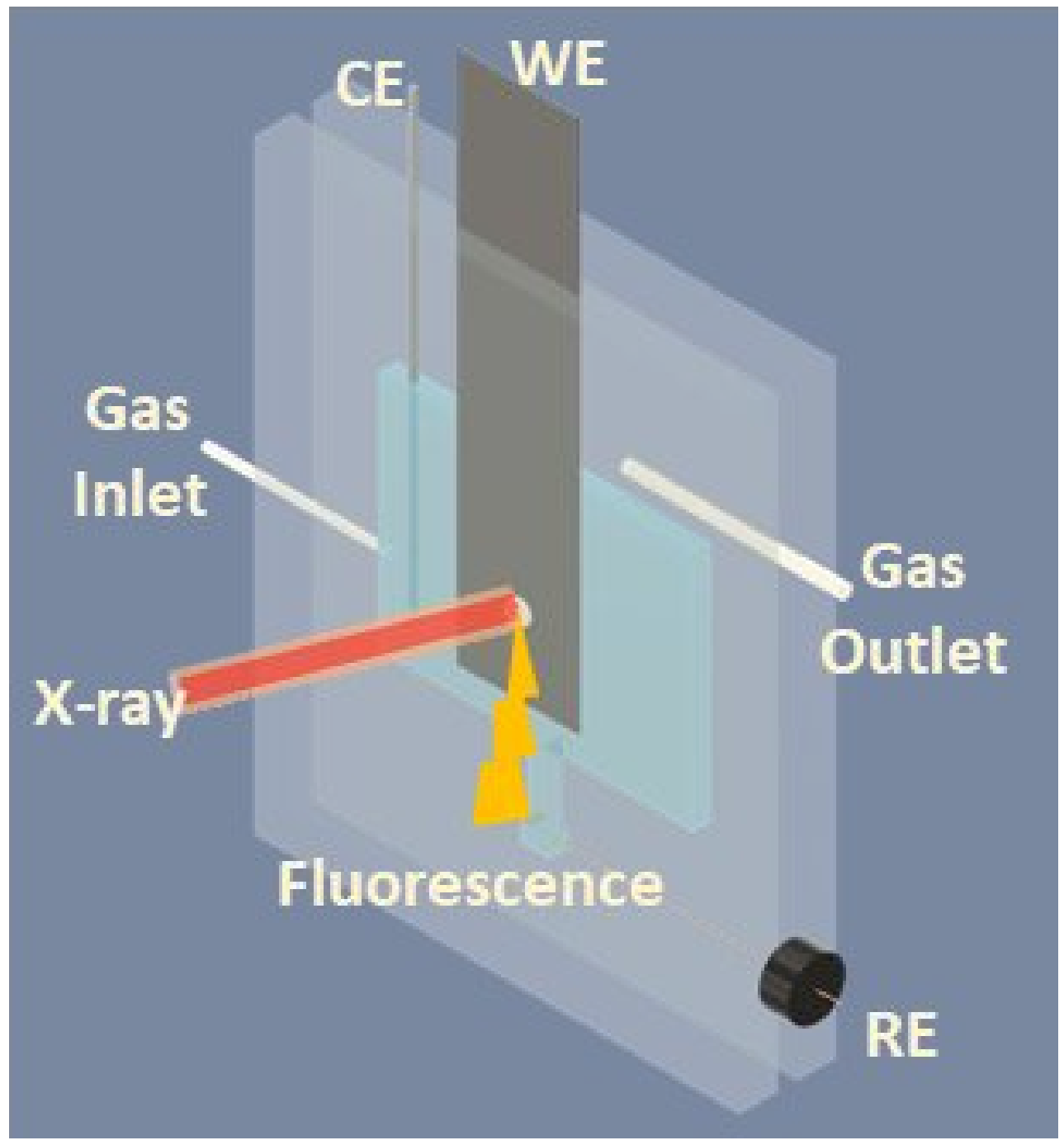

Scheme S1. Schematic illustration of in situ X-ray absorption experiment setup, where WE, CE and RE represent working electrode, counter electrode and reference electrode.

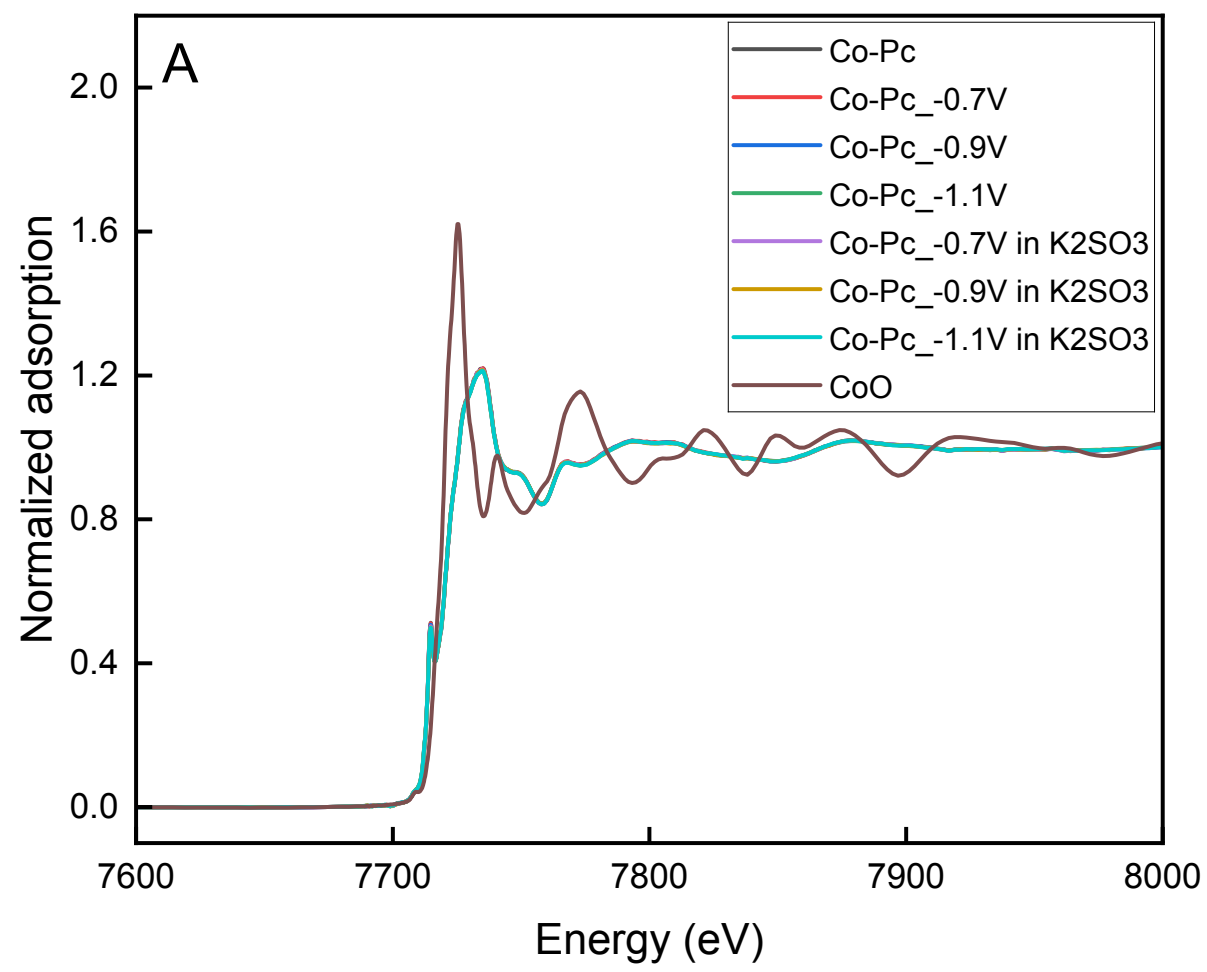

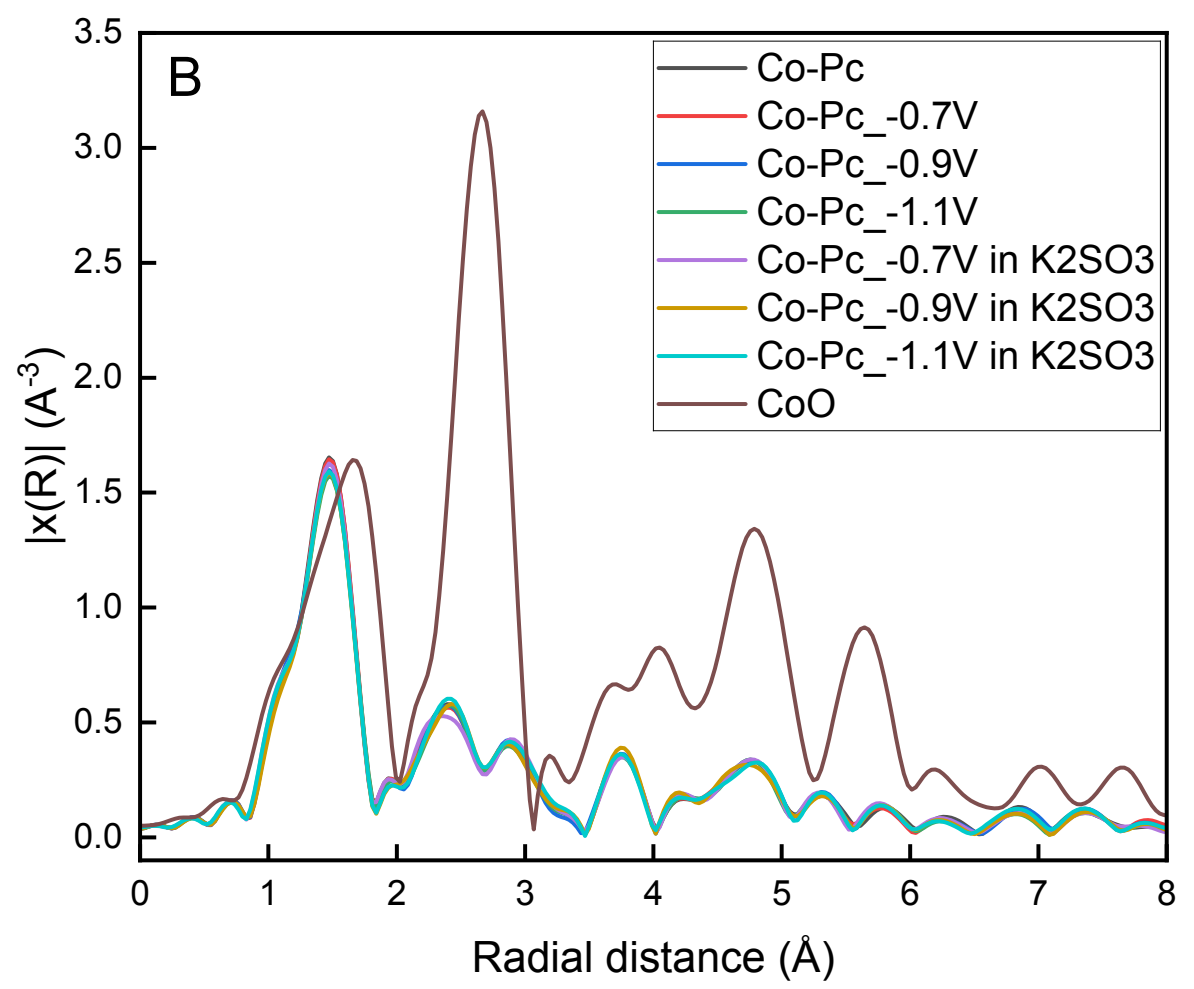

Figure S4. In situ Co K edge XANES spectra (A) and EXAFS spectra (B) of Co-Pc/G at potentials of -0.7, -0.9 and -1.1 V before and after addition of 0.025 M K_2SO_3.

Table S1. In situ EXAFS fitting for Co-Pc/G potentials of -0.7 V before and after addition of 0.025 M K_2SO_3. The fitting k range is 2-12 Å^{-1} and R range is 1-2 Å. The three data points listed in the table were analyzed simultaneously.

**Sample**	**Path**	**N**	**R (Å)**	**σ^2 (Å^2)**	**ΔE (eV)**
Co-Pc-$KHCO_3$	Co-O	4	1.88±0.02	0.000±0.002	5±3
Co-Pc with 0.1M $KHCO_3$	Co-O	4	1.88±0.02	0.000±0.002	5±3
Co-Pc with 0.1M $KHCO_3$ and 0.025M K_2SO_3	Co-O	4	1.88±0.02	0.000±0.002	5±3

Table S2. In situ EXAFS fitting for Cu-Pc/G potentials of -0.7 V before and after addition of 0.025 M K_2SO_3. The fitting k range is 2-8 Å^{-1} and R range is 1-2 Å.

**Sample**	**Path**	**N**	**R (Å)**	**σ^2 (Å^2)**	**ΔE (eV)**
Cu-Pc before reaction	Cu-O	4	1.88±0.02	0.000±0.003	-4±2
Cu-Pc with 0.1M $KHCO_3$	Cu-O	3.8±0.9	1.89±0.03	0.000±0.003	-4±2
Cu-Pc with 0.1M $KHCO_3$ and 0.025M K_2SO_3	Cu-O	3.3±0.6	1.88±002	0.000±0.003	-4±2

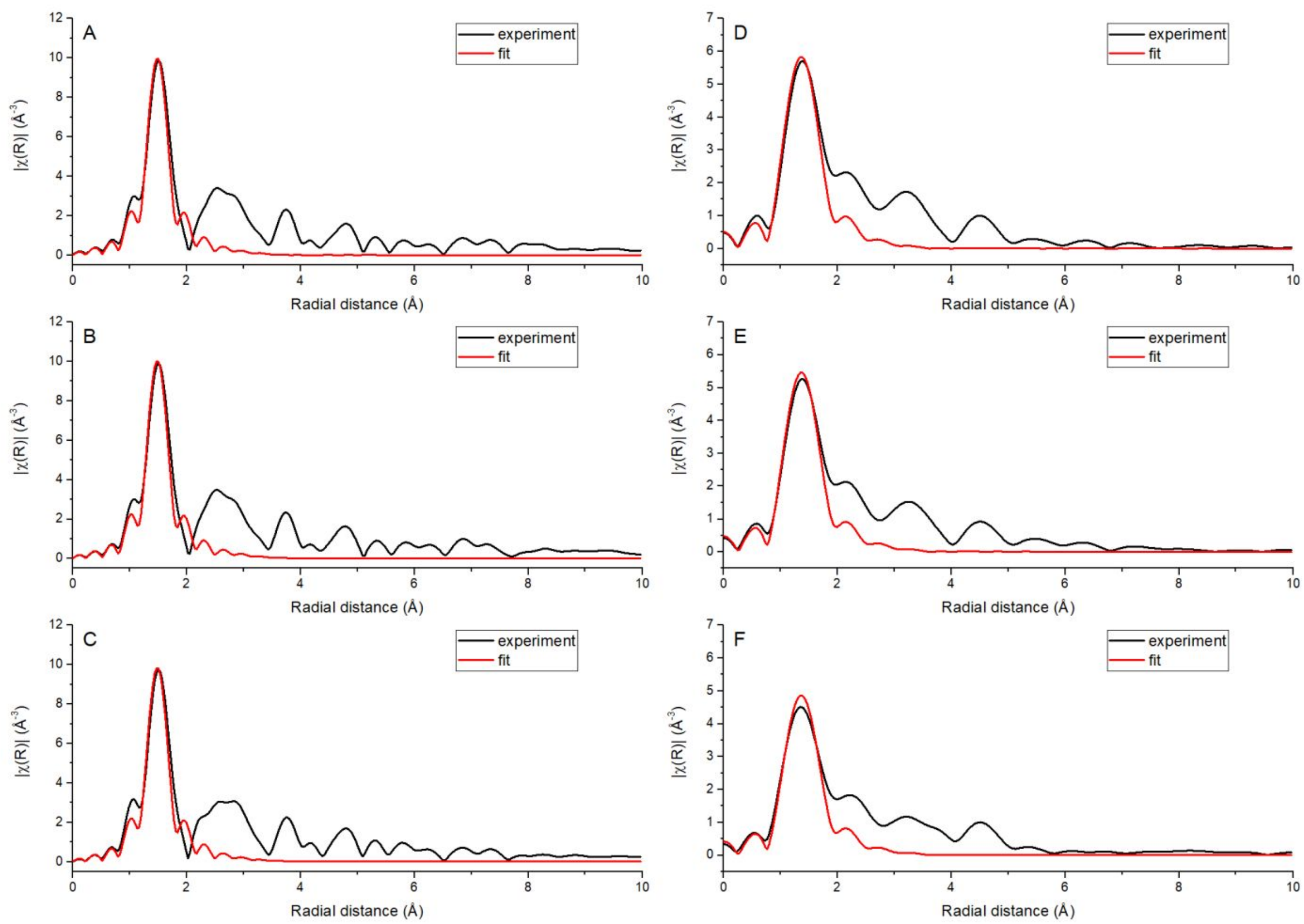

Figure S5. In situ EXAFS spectra R-space fitting for (A) Co-Pc/G before reaction at open circuit potential, (B) Co-Pc/G in 0.1 M $KHCO_3$ at -0.7 V, (C) Co-Pc/G in 0.1 M $KHCO_3$ and 0.025 M K_2SO_3 at -0.7 V, (D) Cu-Pc/G before reaction at open circuit potential, (E) Cu-Pc/G in 0.1 M $KHCO_3$ at -0.7 V and (F) Cu-Pc/G in 0.1 M $KHCO_3$ and 0.025 M K_2SO_3 at -0.7 V.

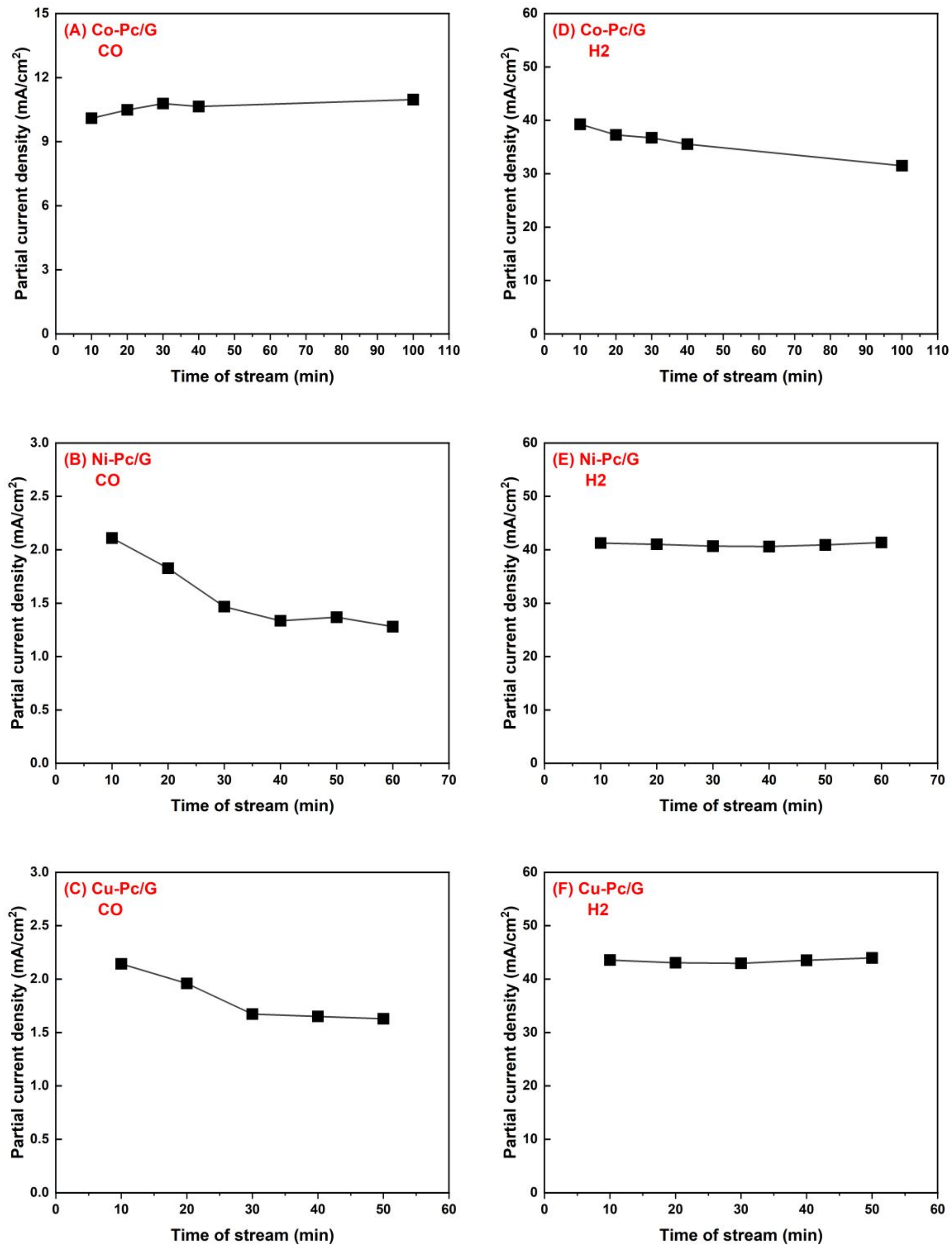

Figure S6. CO partial current density and faradaic efficiency for TM-Pc/G over MEA reactor. A humidified gas mixture of CO_2/Ar (200 ppm balanced by argon) at 2:1 in volume ratio was continuously fed into the cathode side at a flow rate of 30 mL/min, and a 0.1M $KHCO_3$ aqueous electrolyte was circulated on the anode side at a flow rate of 10 mL/min using a peristaltic pump. Chronoamperometry tests were performed at a constant potential of 3.0 V.
